# Comparative analysis of surface-exposed virulence factors of *Acinetobacter baumannii*

**DOI:** 10.1186/1471-2164-15-1020

**Published:** 2014-11-25

**Authors:** Bart A Eijkelkamp, Uwe H Stroeher, Karl A Hassan, Ian T Paulsen, Melissa H Brown

**Affiliations:** School of Biological Sciences, Flinders University, Adelaide, Australia; Research Centre for Infectious Diseases, School of Molecular and Biomedical Science, University of Adelaide, Adelaide, Australia; Department of Chemistry and Biomolecular Sciences, Macquarie University, Sydney, Australia

**Keywords:** Genomics, Virulome, Type VI secretion systems, Membrane

## Abstract

**Background:**

*Acinetobacter baumannii* is a significant hospital pathogen, particularly due to the dissemination of highly multidrug resistant isolates. Genome data have revealed that *A. baumannii* is highly genetically diverse, which correlates with major variations seen at the phenotypic level. Thus far, comparative genomic studies have been aimed at identifying resistance determinants in *A. baumannii*. In this study, we extend and expand on these analyses to gain greater insight into the virulence factors across eight *A. baumannii* strains which are clonally, temporally and geographically distinct, and includes an isolate considered non-pathogenic and a community-acquired *A. baumannii*.

**Results:**

We have identified a large number of genes in the *A. baumannii* genomes that are known to play a role in virulence in other pathogens, such as the recently studied proline-alanine-alanine-arginine (PAAR)-repeat domains of the type VI secretion systems. Not surprising, many virulence candidates appear to be part of the *A. baumannii* core genome of virulent isolates but were often found to be insertionally disrupted in the avirulent *A. baumannii* strain SDF. Our study also reveals that many known or putative virulence determinants are restricted to specific clonal lineages, which suggests that these virulence determinants may be crucial for the success of these widespread common clones. It has previously been suggested that the high level of intrinsic and adaptive resistance has enabled the widespread presence of *A. baumannii* in the hospital environment. This appears to have facilitated the expansion of its repertoire of virulence traits, as in general, the nosocomial strains in this study possess more virulence genes compared to the community-acquired isolate.

**Conclusions:**

Major genetic variation in known or putative virulence factors was seen across the eight strains included in this study, suggesting that virulence mechanisms are complex and multifaceted in *A. baumannii*. Overall, these analyses increase our understanding of *A. baumannii* pathogenicity and will assist in future studies determining the significance of virulence factors within clonal lineages and/or across the species.

**Electronic supplementary material:**

The online version of this article (doi:10.1186/1471-2164-15-1020) contains supplementary material, which is available to authorized users.

## Background

*Acinetobacter baumannii* is a formidable Gram-negative human pathogen that is prominent in hospitals where it is a common cause of infections in critically ill patients in intensive care units and in particular with using respiratory assistance [1]. Clinical *A. baumannii* isolates display major phenotypic differences in virulence-associated phenotypes such as, biofilm formation, adherence to human epithelial cells, invasion, motility and cytotoxicity [2–6].

No doubt, adherence of *A. baumannii* on surfaces of medical devices is critical for its spread within the hospital ward and between patients. A recent study has suggested that equipment such as portable X-ray equipment or wheel chairs can be cross-infected with *A. baumannii* due to aerolization of the organism, thereby negating the need for direct person-to-person contact [7, 8]. Furthermore, colonization of ventilators or catheters can be directly related to the occurrence of pneumonia, urinary tract infections and bacteremia [9, 10]. Ventilators and catheters may become reservoirs for pathogens such as *A. baumannii* as these niches are not necessarily exposed to the antimicrobial agents administered to the patients nor are they accessible during normal hospital cleaning procedures. Certainly, longer hospital stays are likely to induce an increased risk of *A. baumannii* infections as intubation or catheterization results in prolonged exposure of the patient to the pathogen. It has been proposed that a specific form of biofilm known as a pellicle is most relevant in the persistence of *A. baumannii* in environments other than within the host niches. Pellicle formation, is apparent at the liquid/air interface, and is more pronounced at room temperature as compared to incubation at 37°C [11]. It has also been shown that classical biofilms at the solid/liquid interface and pellicle formation are not directly correlated when examining different strains [2].

A critical step in the colonization of host tissues is adherence to eukaryotic cells and can therefore be considered one of the first steps in disease progression. The ability of clinical *A. baumannii* isolates to adhere to biotic surfaces has been investigated in numerous cell culture experiments [3, 12–14]. Major differences in adherence potential exist across clinical isolates, similar to that seen when studying biofilm formation. Variances have also been observed between the level of biofilm formation and the hemagglutination of human group AB erythrocytes by clinical *A. baumannii* isolates [15]. Therefore, a direct correlation between adherence to abiotic and biotic surfaces has not be established in clinical *A. baumannii* isolates [3, 4, 16, 17]. This suggests that *A. baumannii* possesses various independent molecular mechanisms for adherence to distinct surfaces.

A further potential virulence factor is bacterial motility [18–23]; *A. baumannii* participates in at least two forms of motility designated twitching and swarming [3, 6, 23]. In addition to surface-presented protein structures, which will be discussed in more detail below, *A. baumannii* expresses various other macromolecules at the surface that are likely to play a role in persistence and virulence, e.g., lipopolysaccharides and/or capsule. These surface-associated factors play a role in biofilm formation and protection from host-defense mechanisms [24, 25].

Despite the large number of studies published on *A. baumannii* virulence factors, few studies have attempted to determine the conservation of these factors across species, possibly due to significant strain-to-strain variation. Therefore, we examined known and potential virulence factors across eight distinct *A. baumannii* isolates using genome-mining techniques. These factors were subsequently analyzed using comparative genomics to gain greater understanding of their distribution and potential significance within this species.

## Results and discussion

### Strains selected for comparative analyses

The genomic data made available over the last decade have significantly advanced our knowledge of *A. baumannii.* However, the number of studies in which virulence traits are examined at a genetic level is limited and a broad-scale comparative analysis of these traits between strains that differ in their virulence potential is warranted. In our study, a diverse collection of *A. baumannii* strains, isolated across different geographic and temporal regions, was examined to comprehensively assess the surface-exposed components of the *A. baumannii* virulome. Two isolates from the international clone (IC) I lineage (AB0057 and 6870155) and two from the IC II lineage (ACICU and WM99c) were included. To gain greater insight into clonal conservation, the two selected isolates per IC lineage were isolated from different continents (Table 1). The remaining four isolates examined in this study did not group within the defined IC lineages. Three of these are the widely studied strains ATCC 17978, ATCC 19606T and SDF. Additionally, both ATCC 17978 and ATCC 19606T were identified more than 50 years ago, making them appropriate targets to examine temporal differentiation. The *A. baumannii* strain D1279779 was isolated from an outpatient from a remote area in tropical Australia and therefore represents the first fully sequenced community-acquired *A. baumannii* isolate [26], whereas SDF is the only fully sequenced representative of a non-pathogenic isolate.

**Table 1:** **Strain characteristics**

Clonality	Strain	Site of isolation	Country of origin	Reference
IC I	6870155	Sputum	Australia	[3]
	AB0057	Blood	USA	[27]
IC II	WM99c	Sputum	Australia	[3]
	ACICU	Cerebrospinal fluid	Italy	[28]
Non-IC	ATCC 17978	Meninges	France	[29, 30]
	ATCC 19606T	Urine	USA	[31]
	D1279779	Blood	Australia	[26]
	SDF	Louse	France	[32]

**Figure 1:**
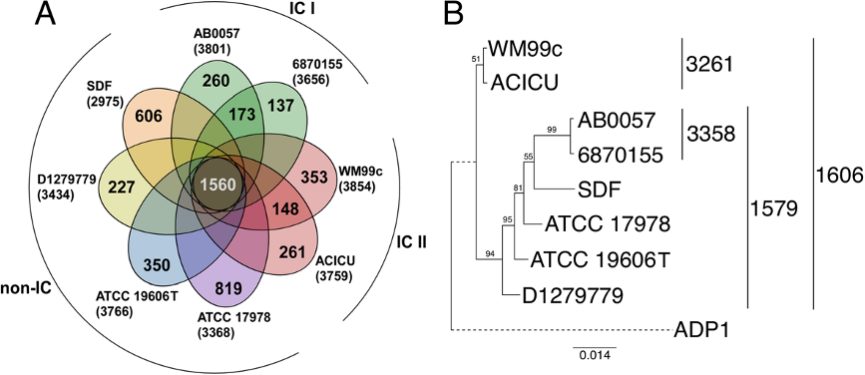
**Genomic diversity among** ***Acinetobacter*** **strains. (A)** Partial Venn diagram. Each *A. baumannii* strain is depicted by an oval colored according to IC designation. Numbers presented in overlapping regions of the ovals show the number of genes shared by that group of strains. For example, the core genome size shown in the center, where all ovals overlap, is 1560 ORFs. Numbers in the non-overlapping regions show the number of genes unique to that strain. **(B)** Phylogenetic tree based on a concatenated alignment of the core housekeeping genes *cpn60*, *fusA*, *gltA*, *pyrG*, *recA*, *rplB* and *rpoB* in the strains under investigation. *A. baylyi* ADP1 (GenBank; CR543861) was used as an outgroup and the dotted line used in the ADP1 branch indicates a branch length greater than that shown. Numbers to the right of the tree show the size of the total number of conserved ORFs (the core genome) in the set of strains indicated.

A genome-wide analysis of the shared gene content was performed which showed that 1560 genes were shared between all eight strains, which can be considered the “core genome”. However, strain SDF has undergone major genomic rearrangements and the remaining seven strains have an additional 503 shared genes, hence, 2063 genes were shared between the virulent strains included in this study. In respect to divergence from the core genome (1560 genes), strain ATCC 17978 was found to possess the highest number of unique genes (*n* = 819) followed by the SDF isolate (*n* = 606) (Figure 1A). The direct genome comparison revealed that clonality, examined as described previously [33], correlated with their total shared gene content; isolates within clonal groups showed the highest percentage of shared genes as compared to strains from different clonal groups (Figure 1B; Additional file 1). Strain SDF shared the lowest number of genes with other isolates. However, since SDF has a small genome that has undergone considerable genome reduction (only ~3050 open reading frames) it still shared an average of approximately 71% of its coding content with the other isolates used in this analysis. Strain ATCC 17978 also showed comparatively low numbers of shared genes, potentially resulting from its temporal and/or geographic separation from the other strains. The highest proportion of shared genes was 92% between the two IC I isolates AB0057 and 6870155. Overall, these diverse strains exemplify an excellent representation of *A. baumannii* isolates for tracking the spread of virulence determinants, by including clonal strains commonly isolated worldwide (the IC strains), a community-acquired strain and strains that are geographically and temporally distant. The avirulent isolate SDF was predominantly included for comparative purposes.

### Type I pili

**Figure 2:**
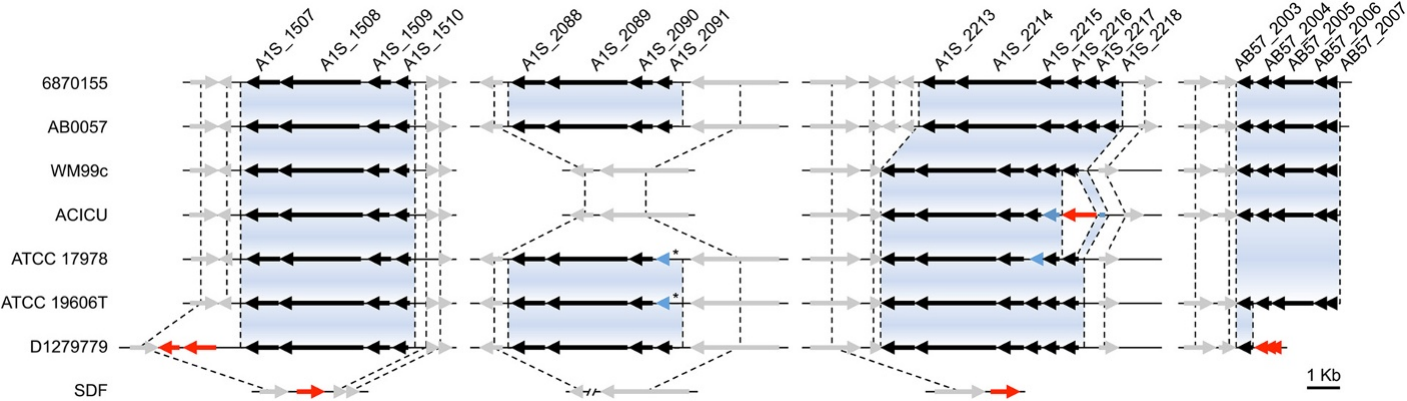
**Genetic analysis of the Type I pili clusters.** Genomic organization of the Type I pili clusters A1S_1507-1510, AB57_2003-2007, A1S_2088-2091 and A1S_2213-2218 (Csu-cluster) in ATCC 17978 and comparison to those in other strains. Genes have been drawn to scale. The arrows represent ORFs and depict the direction of transcription; pili components (black), adjacent homologous genes (grey), transposases (red) and inactivated genes (blue). The blue shading indicate a high level of homology between genes from different strains (E-value <10^-30^). The asterisks indicate the location of a polymeric tract seen in A1S_2091 and its ortholog in strain ATCC 19606T.

One of the most common protein structures decorating the outer surface of pathogens are the Type I pili, which often play a major role in adherence of Gram-negative pathogens, and are well documented in another member of the gamma-proteobacteria, uropathogenic *Escherichia coli* [34]. Four gene clusters encoding these pili have been identified in *A. baumannii* (Figure 2), which include the functionally characterized *csu*-cluster (A1S_2213-2218) [35–37]. Interestingly, the *csu*-cluster is likely to be non-functional in two strains; in ACICU as a result of an insertion, and in ATCC 17978 due to a single nucleotide polymorphism resulting in truncation of *csuB*, as previously described [3]. At a proteomic level, the P pili annotated proteins (cluster AB57_2003-2007), CsuC and CsuD, and putative Type III pili (Table 2; A1S_0690-0695), were found to be highly expressed in cells in the pellicle [38] perhaps highlighting their role in this phenotype.

**Table 2:** **Comparative analysis of surface-presented protein structures**

Gene/locus-tag	Gene product/function	6870155	AB0057	WM99c	ACICU	ATCC 17978	ATCC 19606T	D1279779	SDF
A1S_1032	Autotransporter adhesin	-	-	+	+	+	+	+^*^	-
A1S_2696	Biofilm associated protein	+	+	+	+	+	+	-	+
AB57_3081	Biofilm associated protein	+	+	+	+	-	+	-	+^*^
A1S_2840	OmpA	+	+	+	+	+	+	+	+
A1S_0884	OmpA-like	+	+	+	+	+	+	+	+
A1S_1033	OmpA-like	-	-	+	+	+	+	+	-
A1S_1193	OmpA-like	+	+^*^	+	+	+	+	+	+
A1S_0108	Pmt	+	+	+	+	+	+	+	+
A1S_0269	Type II secretion system	+	+	+	+	+	+	+	+
A1S_0690-0695	Putative Type III pili	+	+	+	+	+	+	-	+

^*^Pseudogenes.

+Gene present.

-Gene absent.

Gene clusters orthologous to A1S_1507-1510 (fimbrial genes) are well conserved across the seven virulent *A. baumannii* strains included in this study (Figure 2). Interestingly, this cluster was found to be down-regulated in strain ATCC 17978 under iron-limitation [39], which has adverse effects on pellicle formation (Eijkelkamp *et al.*, unpublished data). Furthermore, we have recently examined an ATCC 17978 derivative, which carries an insertionally-inactivated copy of the major global regulator H-NS [22]. Increased expression of A1S_1507-1510 and enhanced pellicle formation was noted in this mutant strain [22]. A recent study by Rumbo-Feal *et al.* examining the expression of genes in biofilm versus planktonic cells in strain ATCC 17978 has shown that A1S_1507 is highly up-regulated in biofilm cells compared to planktonic cells as were the *csu* genes *csuD*, *csuC* and *csuA/B* [40]. Therefore, Type I pili are likely to play a role in *A. baumannii* adherence and biofilm/pellicle formation, however, the exact contribution of these three clusters under different conditions requires further examination.

The role of the A1S_2088-2091 Type I pili cluster in *A. baumannii* virulence has not been established. The IC II isolates appear to lack this cluster and the genomic region in strain SDF appears to have undergone major rearrangements (Figure 2). Furthermore, Sanger sequencing confirmed sequence length variation in a polymeric tract at the 5′-end of A1S_2091 which has rendered this cluster inactive in ATCC 17978 and ATCC 19606T [3]. The poly-thymine tract found in the 5′-end of A1S_2091 may represent the first phase-variation mechanism of *A. baumannii*. During replication, the probability of ‘slippage’ in polymeric tracts can be relatively high [41], which may result in a change of the A1S_2091 open reading frame (ORF).

### Type IV fimbriae

The Type IV fimbriae (TFF) are large protein complexes with structural components in both the inner and outer membranes. Whereas Type I pili have been implicated in biofilm formation, cell aggregation and attachment, the TFF have been shown to be involved in motility [23, 42] and are likely to play a role in virulence in *A. baumannii*, although, this has not been proven to date. Unlike earlier reports on the absence of *pilX*, *pilV* and *pilT* of the TFF in strain ATCC 17978 (Table S2 in Antunes *et al.* [43]), we found that strain ATCC 17978 possesses all orthologous genes identified, in for example, strain ACICU. In fact, SDF was found to be the only strain lacking various TFF genes as a result of insertional disruption/deletion events, corroborating the findings by Antunes and colleagues. Notably, the A1S_3166 (*pilE*) ortholog is truncated in strain ATCC 19606T as a result of an adenosine deletion in a position that correlates to 3,648,940 in strain ATCC 17978. Whether this potential nucleotide deletion has phenotypic consequences remains to be examined. Despite the presence of the individual genes required for TFF biosynthesis in most strains, there are significant differences in the major fimbrial subunit (PilA) between strains. We have previously shown that PilA, is highly variable between strains and that this correlates with differences in their motility characteristics [3].

### Type V secretion systems

The significance of Type V secretion systems, also called autotransporter proteins, in bacterial pathogenesis has been well documented [44–46]. The autotransporter Ata from *A. baumannii* strain ATCC 17978 has been shown to play an important role in adherence and virulence [47]. Our analyses indicated that this might be the only autotransporter in *A. baumannii*. Homologs of Ata (A1S_1032) were only found in the IC II strains WM99c and ACICU, and in the non-IC strains ATCC 17978, ATCC 19606T and D1279779 (Table 2). However, Ata is most likely non-functional in strain D1279779 as the ORF has been truncated significantly at the 5′-end. Major sequence variation was seen in the central region of this gene; what impact this may have on virulence is unknown. The autotransporter gene appears to be co-transcribed with a gene encoding a putative OmpA-like protein (A1S_1033). We recently showed that these two genes are also regulated by H-NS [22]. H-NS is known as a xenogeneic silencer, which suggests that *ata* and A1S_1033 may have been acquired horizontally, which is corroborated by the finding that the IC I strains do not harbor these potential virulence factors. Bentancor and co-workers identified *ata* in approximately 58% of clinical isolates, unfortunately the IC type of the strains was not described so it is not possible to conclude whether *ata* is restricted to certain clonal linages from this analysis [47]. Major differences in expression of *ata* were observed, and although *ata* plays a role in biofilm formation and is expressed at high levels in strain ATCC 17978 [47] we have previously shown that this isolate produces a poor biofilm as compared to other *A. baumannii* strains [3]. The exact role Ata plays in virulence and adherence in strains other than ATCC 17978 has yet to be elucidated.

### Type VI secretion systems

The Type VI secretion systems (T6SSs) are known to be involved in cell invasion and competition amongst bacteria and have been identified in a number of pathogens [48–50]. T6SSs have also been proposed to play a role in Type I pili regulation and inter-bacterial communication [51–53]. Their ubiquitous presence in both pathogenic and non-pathogenic bacteria also shows that these phage-related protein structures are involved in more functions than solely pathogenicity [48, 54]. The function of T6SS in *A. baumannii* has been reported in a limited number of studies. An ATCC 17978-derived T6SS mutant was found to be uncompromised in virulence and bacterial competition studies [55]. However, in a second publication examining the role of T6SS in bacterial competition, *Acinetobacter nosocomialis* strain M2 was found to utilize the T6SS for killing of *E. coli* [23, 56] potentially giving strains expressing T6SSs a competitive advantage.

The gene cluster encoding the *A. baumannii* T6SS was identified in all strains included in this study, except for the community-acquired isolate D1279779. Furthermore, part of the T6SS cluster in the avirulent strain SDF (ABSDF2238-2242) displayed low levels of sequence similarity with orthologs in the other *A. baumannii* strains (data not shown), which may result in functional differentiation of the T6SS in strain SDF, as has been illustrated in *Pseudomonas aeruginosa* [54, 57]. The common *P. aeruginosa* reference strain, PA01, harbors three T6SSs with distinct functions; these being eukaryotic cell invasion and inter-bacterial communication/competition [54, 57]. Comparative analysis of the *A. baumannii* T6SS to the functionally defined T6SSs from *P. aeruginosa* did not show higher sequence similarity between particular members, hence, the function of the T6SSs in the strains examined here remains to be determined experimentally.

**Figure 3:**
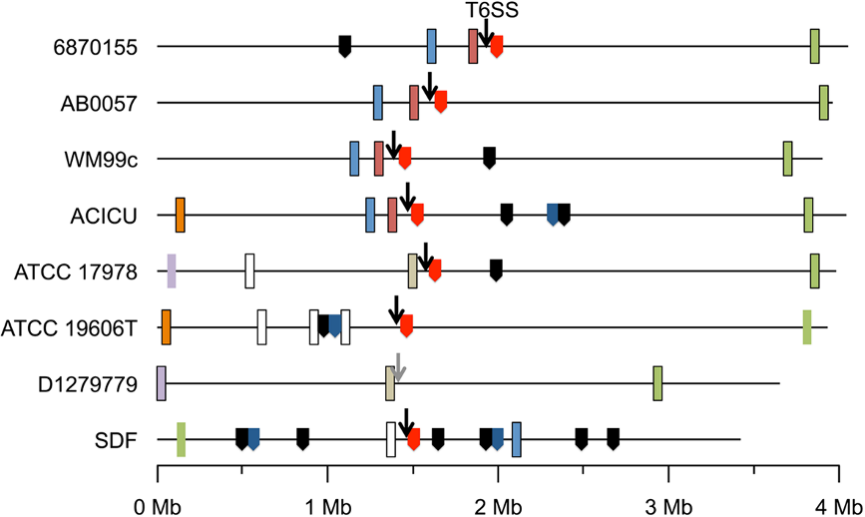
**Genomic positioning of genes encoding components of the Type VI secretion system.** The complete genomes (horizontal black lines) have been shown to scale. The black arrows indicate the position of the cluster encoding the type VI secretion system; this cluster is not present in strain D1279779 with the grey arrow pointing to its predicted position. The rectangles indicate *vgrG*-like genes, their genomic position and the color show orthology (E-value <10^-30^). No significant similarity was seen between those represented in white. Rectangles without borders represent disrupted *vgrG*-like genes. The genomic position of genes encoding PAAR-repeat domain proteins has been indicated by the block-arrows. The color indicates to which class the encoded PAAR-repeat domain belongs; class 1 (red), class 2 (black) and class 5 (blue).

Three different types of putative effectors of the *A. baumannii* T6SSs have been identified; Hcp (hemolysis co-regulated protein), which is encoded by the highly conserved gene A1S_1296 (co-expressed with the other structural components of the T6SS), and a multitude of Valine-Glycine-Repeat protein G-like (VgrG-like) and Proline-Alanine-Alanine-Arginine (PAAR)-repeat domain proteins. Despite the high levels of homology observed between most T6SS clusters, the genes encoding the VgrG and PAAR-repeat domain proteins were found to be scattered throughout the genomes and only a few of these were found to be conserved between strains (Figure 3). The scattering of *vgr*-like genes is not unusual and has been documented in other bacteria such as *Vibrio cholerae* and *E. coli* where the *vgr*-like genes were initially thought to be accessory components of the recombination hot spot family [58, 59]. One of the genes annotated as a ‘Vgr-related’ protein encoded by A1S_3364 in strain ATCC 17978 was identified in all eight *Acinetobacter* strains, however, the C-terminal domain is highly variable. Furthermore, a major truncation of the gene encoding this VgrG protein appears to have occurred in strains ATCC 19606T and SDF, which likely renders it inactive. A number of *vgrG* genes were also identified in close proximity to the genomic position of the T6SS itself (Figure 3), which is not unusual and has been observed in numerous other bacteria [50]. However, the exact location of this gene, represented by A1S_1288 in strain ATCC 17978, differs and homologs can only be found in strains 6870155, AB0057, WM99c and ACICU. Overall, our comparative analyses indicated that at least 11 unique insertion events of *vgrG* have taken place in the eight *A. baumannii* strains included in this study (Figure 3). Most strains harbor three full-length *vgrG* genes, including D1279779, which does not possess the T6SS itself; ACICU possesses four.

A recent report on PAAR-repeat proteins demonstrated their significance in T6SS-mediated bacterial competition [60]. The PAAR-repeat domain was found to dock onto the terminus of the pilus-like structure formed by VgrG, sharpening the T6SS complex and enabling penetration of host cells. Schneider and co-workers have subdivided the PAAR-repeat proteins into seven different classes, depending on the presence of additional C- and/or N-terminal domains. We identified class 1, 2 and 5 PAAR-repeat proteins in the *A. baumannii* genome (Figure 3 and Additional file 2), all of which showed highest homology to the pfam05488 type of PAAR-repeat domains (data not shown). The class 1 PAAR-repeat proteins contain no additional domains and the genes encoding these in *A. baumannii* were predominantly found clustered with the other components of the T6SS system, such as A1S_1306 in strain ATCC 17978. A total of 12 class 2 PAAR-repeat proteins were identified, with strain SDF harboring seven, of which six were identified on the chromosome and one on plasmid p2ABSDF (Additional file 2). The proteins within this class possess two domains, the PAAR-repeat domain positioned N-terminally and in case of the *A. baumannii* class 2 proteins a C-terminal domain of unknown function. Only strains ATCC 19606T, ACICU and SDF possess a member of the class 5 PAAR-repeat proteins. These proteins are composed of three domains with the PAAR-repeat domain centralized. Unfortunately, none of the N- and C-terminal extensions of this class in *A. baumannii* encode domains with known functions. The previously characterized PAAR-repeat proteins ACIAD2681, ACIAD0052, ACIAD0051 from *Acinetobacter baylyi* ADP1 [60] were found to cluster within class 1, 2 and 5, respectively (Additional file 2). There are major differences in the number of PAAR-repeat proteins between the strains in this study, as none were identified in strain D1279779 and SDF was found to possess 10, representing members of three distinct classes. The absence of genes encoding the PAAR-repeat containing proteins in strain D1279779 correlates with the lack of the structural components of the T6SS.

We have recently shown that H-NS regulates expression of the T6SS cluster [22], however, the whole transcriptome analysis also indicated that the genes encoding VgrG-like proteins or PAAR-repeat proteins are not under regulatory control of H-NS. Further work is required to elucidate the function of the different types of VgrG-like proteins and PAAR-repeat proteins in *A. baumannii*.

### Additional membrane-associated protein structures

Protein or toxin secretion systems play an important role in pathogenesis [61] and in order for proteins to be secreted they need to cross both the cytoplasmic and outer membrane of *A. baumannii*. We identified various genes encoding complex protein structures that could mediate this process (Table 2). Unfortunately, to date the characterization of these systems in *A. baumannii* has been limited. Screening of a transposon library, revealed that insertions in A1S_0269 abrogated protection during growth in human serum [62]. An insertion in A1S_0269 may have polar effects on the co-transcribed genes A1S_0270 and A1S_0271. The A1S_0269 gene is predicted to produce the N component of a putative Type II secretion system which has been shown to be non-essential for Type II secretion [63], A1S_0270 encodes part of the inner membrane platform and A1S_0271 encodes the outer membrane secretin/channel. Exactly how protection to human serum is mediated requires further characterization. We found the three components of this putative Type II secretion system to be highly conserved between all *A. baumannii* strains included in this study (Table 2). However, an insertion sequence has disrupted the upstream region of this cluster in strain SDF.

The role of the outer membrane protein OmpA (A1S_2840) in *A. baumannii* virulence has been studied intensively [12, 64–67] and has been shown to facilitate adherence to eukaryotic cell surfaces and cell invasion [12, 65]. Furthermore, OmpA promotes cell death of lung epithelial cells by induction of interleukin-8 and other cytokines [14, 68, 69]. OmpA was found to be well conserved amongst all eight isolates. Three additional *ompA*-like genes were identified in the *A. baumannii* genomes (A1S_0884, A1S_1033 and A1S_1193). Both A1S_0884 and A1S_1193 were identified in all eight strains, however, a frame-shift in AB57_1330 may render this *ompA*-like gene inactive in strain AB0057 (Table 2). As described above, A1S_1033 appears to be co-transcribed with the autotransporter *ata* and was identified only in the IC II strains, WM99c and ACICU, and in strains ATCC 17978, ATCC 19606T and D1279779.

The biofilm associated protein (Bap) facilitates adherence in *A. baumannii* [70–72] and is likely to be involved in virulence as shown in other pathogens [73]. This large protein shows major sequence variation, which has previously been described in detail [70, 72]. The *bap* gene in strain ACICU appears to have been annotated as a number of distinct ORFs and requires sequence confirmation. Furthermore, Loehfelm *et al.* [71] described the identification of two distinct Bap-like proteins in strain ATCC 17978 and genome sequence analysis indicated that the genes encoding these fragments, A1S_2724 and A1S_2696, may actually have been separated by rearrangement events [71]. A second Bap-like protein encoded by AB57_3081 in *A. baumannii* strain AB0057 can also be identified in strains 6870155, WM99c, ACICU and ATCC 19606T (Table 2). An insertion sequence leads to truncation of this protein in strain SDF, whereas strains ATCC 17978 and D1279779 do not possess any sequences homologous to AB57_3081. The community acquired isolate D1279779 appears to encode no Bap or Bap-like proteins (Table 2).

The major facilitator superfamily (MFS) transporter Pmt has been shown to play a role in adhesion and biofilm formation [74]. Analysis of *pmt* expression revealed that it was elevated in cells in the biofilm when compared to their planktonic counterparts. Furthermore, expression of *pmt* in *E. coli* DH5α cells increased adherence to abiotic and biotic surfaces clearly indicating that this protein plays a role in adherence and, as such, is likely to function in virulence [74]. The mode of action is thought to involve secretion of extracellular DNA which has previously been reported for *A. baumannii* [74]. The *pmt* gene (A1S_0108) was identified in all strains regardless of their virulence potential or their ability to form biofilms (Table 2).

### Capsule biosynthesis

**Figure 4:**
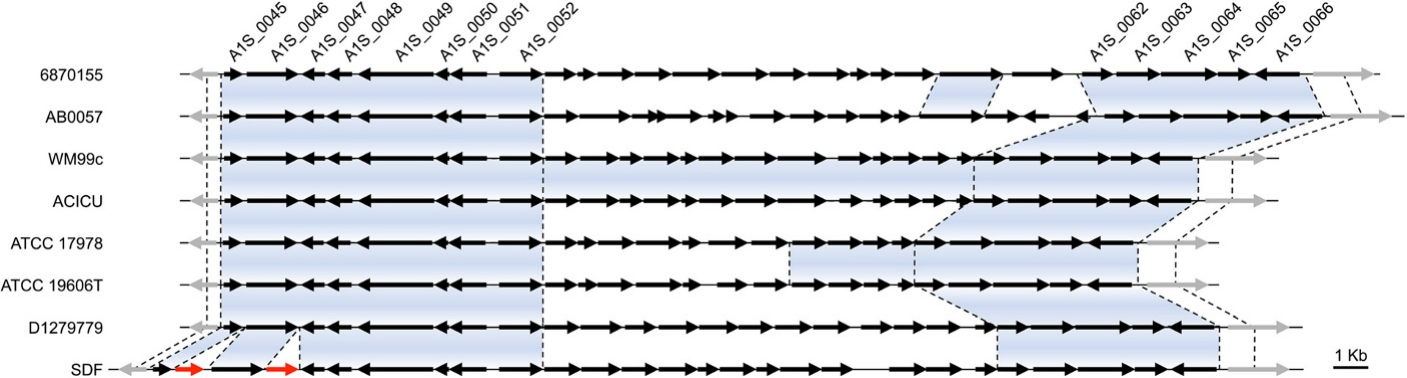
**Genomic region involved in production and secretion of capsule.** Genomic organization of the region involved in capsule biosynthesis. Genes have been drawn to scale. The arrows represent ORFs and depict the direction of transcription; capsule biosynthesis genes (black), adjacent homologous genes (grey) and transposases (red). The blue shading indicates a high level of homology between genes from different strains (E-value <10^-30^).

The O antigen and/or the capsule have long been recognized as essential virulence factors in numerous bacterial species as mutants that are either rough (contain no O antigen) or acapsular are generally avirulent [75]. By definition, O antigen is linked to the outer core of the lipopolysaccharide (LPS), which constitutes the outer leaflet of the outer membrane in Gram-negative organisms, whereas a capsule is generally considered to be unlinked or linked to the cell wall. There is mounting evidence that *A. baumannii* produces capsule [76–79], because the *waaL* gene is lacking within the surface polysaccharide operon. WaaL is required for linking O antigen to the outer core, thus these strains are likely to produce capsule and not O antigen. A *waaL*-like gene present elsewhere in *A. baumannii* genomes was thought to be involved in protein glycosylation and not O-antigen linkage [80]. However, Wright *et al.* [81], has suggested that a second *waaL* gene present in some strains of *A. baumannii* may actually be involved in O-antigen linkage as this second gene does not have the domain thought to be required for protein glycosylation [81]. Examination of the strains in our study showed that only the EC II clone strains (ACICU and WM99c) and ATCC 19606T and 6870155 possessed homologs of this second *waaL*-like gene (Table 3). Comparison of the genome organization of ATCC 17978 encompassing ORFs A1S_0049 to A1S_0066 (Figure 4; Table 3) with the other seven strains used in this study indicates at least six different genetic arrangements of this region, which would imply six different capsule types. This supports the notion that many distinct cluster types may be present across the species [76, 77, 79]. At the start of this region there are four conserved ORFs (A1S_0049-0052). Adjacent to these highly conserved genes is a genetic region of higher variability, encoding proteins involved in the biosynthesis of specific activated sugar precursors and transferases for each of the capsule types. The last section of this locus contains a second conserved region of five ORFs (A1S_0062 to A1S_0066).

**Table 3:** **Comparative analysis of surface decoration and secretins**

Gene/locus-tag	Gene product/function	6870155	AB0057	WM99c	ACICU	ATCC 17978	ATCC 19606T	D1279779	SDF
A1S_3792-2160-2162	PNAG	+	+	+	+	+	+	+	-
A1S_0938-0940	*pga*-like	+	+	+	+	+	+	+	-
A1S_2752	PmrC	+	+	+	+	+	+	+	-
ACICU_01072	Phosphoethanolamine transferases	-	-	+	+	-	-	-	-
ABD1_10510	Phosphoethanolamine transferases	-	-	-	-	-	-	+	-
ACICU_03379	WaaL-like	-	-	+	+	-	+	+	-
A1S_0112-0119	Polyketide/lipopeptide biosynthesis	+	+	+	+	+	+	+	-

+Gene present.

-Gene absent.

The enzymes involved in the biosynthesis of the various precursors and activated sugars closely match the requirements for the surface polysaccharide structure determined for *A. baumannii* [82–84]. A recent publication by Kenyon and Hall (2013) has identified nine genetically distinct capsule loci in *A. baumannii* designated KL1-KL9 and includes a more detailed analysis of the various regions within these loci beyond what is discussed here. The study by Hu *et al.* [79] has extended this to 25 well defined serovars and followed from an earlier study examining 152 *A. baumannii* isolates using polyclonal antisera which revealed that there are possibly over 20 distinct surface polysaccharide types in this organism [85]. Thus, there appears to be significant variation in both the genetic content and arrangement of the proposed capsule loci. Furthermore, examination by Hu *et al.* [79] of the capsule operon of other fully or partially sequenced *A. baumannii* genomes found significant differences in the *wzy* (polymerase) and *wzx* (translocase) genes indicating that there are significantly more serovars than the 25 well described types [79]. In *Acinetobacter* there are currently 77 distinct polysaccharide gene clusters not all of which have to date been identified in *A. baumannii*. No doubt further sequencing may reveal details of these other serovars.

### Lipid A modifications

The lipid A fraction of LPS is considered a major virulence factor of Gram-negative bacteria. In *A. baumannii* lipid A has predominantly been studied for its role in resistance to the polymyxin antimicrobial peptides [86–90]. Interestingly, the modifications of lipid A that are associated with polymyxin resistance may negatively affect the virulence potential, a topic currently under debate [91, 92]. Lipid A modification occurs as a result of up-regulation of *pmrC* (A1S_2752), a gene encoding a lipid A phosphoethanolamine transferase. We found several other genes encoding lipid A phosphoethanolamine transferases in the genomes examined in this study, which may affect antigenicity of this membrane component and/or polymyxin susceptibility. Whereas *pmrC* is highly conserved across all genomes with the exception of strain SDF, the additional phosphoethanolamine transferases were found to be most often associated with horizontally-acquired regions. For example, ACICU_01072 was found in a large phage-related cluster in the IC II (ACICU and WM99c) strains only. The community-acquired strain D1279779 was found to possess a second distinct phosphoethanolamine transferase (Table 3).

### Other carbohydrate-based surface decorations

The *A. baumannii* strains included in this study appear to possess two clusters encoding proteins required for biosynthesis of poly-β-1-6-*N*-acetylglucosamine (PNAG), a polysaccharide that is critical for biofilm formation. Only one of the gene clusters (A1S_3792, A1S_2160-2162) has been functionally examined [25]. As observed with the characterized cluster, the second cluster (A1S_0938-0940) is highly conserved across most strains; both clusters have been removed by insertion-deletion events in strain SDF (Table 3).

A large gene cluster (A1S_0112-0119) believed to be responsible for the production of a biosurfactant, most likely in the form of lipopeptides, has been shown to play a role in surface motility [42]. Intriguingly, expression of this cluster is affected by differing levels of quorum-sensing signals [42], the global regulatory protein H-NS [22] and cyclic-AMP (Giles *et al.*, unpublished data). This cluster and the DNA-binding site for the quorum-sensing regulator AbaR was identified in all seven virulent strains, however, there is major sequence variation in other parts of the upstream region (data not shown), which could result in differential repression by H-NS.

## Conclusions

In this study we sought to examine the surface-exposed virulence factors of the human pathogen *A. baumannii*. We described for the first time a range of novel prospective virulence candidates, including a fourth Type I pili cluster (A1S_2088-2091), the VgrG-like proteins, the PAAR-repeat domain proteins, Bap-like proteins and various OmpA-like proteins. Furthermore, we identified additional phosphoethanolamine transferases and a gene cluster putatively involved in the production of PNAG (A1S_0938-0940). All of these candidates make excellent targets for experimental examination.

Our genome comparison data show that many of the highly conserved *A. baumannii* virulence genes have been insertionally-disrupted or mutated in strain SDF, providing insight into its non-pathogenic phenotype. As reported previously by our group when studying carbon and nitrogen utilization and drug resistance [26], the community-acquired *A. baumannii* isolate D1279779 was found to be quite distinct from its nosocomial counterparts. Accordingly, in this study we found that strain D1279779 possessed significantly fewer virulence genes. Thus far, the link between the virulence potential of community-acquired *A. baumannii* strains, and the virulence and drug resistance genes has not been fully explored. Multiple horizontally-acquired genomic regions found in the common IC clone strains were also identified in strain D1279779, such as the OmpA-like protein encoded by A1S_1033. This indicates that although geographically isolated, community-acquired isolates appear to be capable of acquiring novel virulence genes. Alternatively, these genes could have been acquired by lateral gene transfer by a common ancestor before the split of the strains. In conclusion, our study shows that the common nosocomial clones have a broad and diverse repertoire of virulence genes, which in combination with their extensive range of resistance genes provides the genotype fundamental to the success of this species.

## Methods

### Genome mining and comparative analyses

Identification of orthologous genes/proteins across the *A. baumannii* genomes was performed using blastp searches (E-value <10e^-30^) in either NCBI [93] or RAST [94]. All eight genomes (GenBank accession numbers can be found in Additional file 1) were aligned using Mauve [95], which was subsequently used for determining genetic positioning and identification of insertion events. The genomic alignment from Mauve was also utilized as a template for generating Figures 2 and 4.

The putative proteomes of the eight strains included in this study were compared using blastp 2.2.25+. Putative orthologs were assigned as reciprocal best hits with an E-value <10e^-5^ and length match greater than 75%.

### Phylogenetic analysis of PAAR-repeat domains

The proteins containing PAAR-repeat domains were aligned using ClustalOmega [96]. Only the PAAR-repeat domains were included for generation of the phylogenetic tree using the Neighbor-Joining method (100 bootstrap analysis replicates) [97]. The tree was visualized in CLC Sequence Viewer 6 (CLC bio, Cambridge, MA, USA).

## Electronic supplementary material

Additional file 1: **Pairwise gene conservation.** The table shows the percentage of genes shared between strains, using a pairwise comparison. The comparison between the IC I isolates has been highlighted in green and between the IC II isolates in red. Also listed are the GenBank accession numbers. (XLSX 49 KB)

Additional file 2: **Phylogenetic analysis of the PAAR-repeat domain containing proteins.** The extension sequences of the class 2 and 5 PAAR-repeat proteins in this analysis were excluded to compare the PAAR-repeat domains only, i.e. based on the class 1 PAAR-repeat proteins. The *A. baylyi* APD1 PAAR-repeat proteins have been included for comparative purposes. The proteins originate from the following strains; _WM99c, WM99c; A1S_, ATCC 17978; HMPREF0010_, ATCC 19606T; ACIAD, ADP1; ACICU_, ACICU; AB57_, AB0057; ABSDF, SDF; and _6870155, 6870155. The different colors indicate the class to which the highlighted clade belongs, class 1, 2 or 5 as described previously [60]. (TIFF 6 MB)
