# Supplementary figures and images for: Comparative analysis of surface-exposed virulence factors of Acinetobacter baumannii
Source: BMC Genomics. 2014 Nov 25;15(1):1020. doi: 10.1186/1471-2164-15-1020 (PMC4256060; doi:10.1186/1471-2164-15-1020)

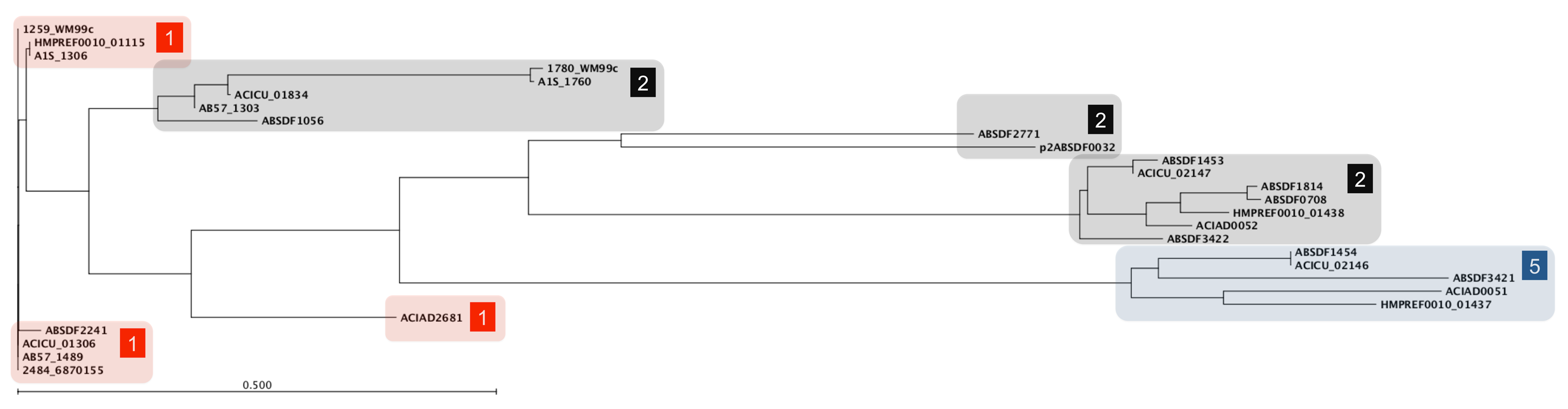

Supplement: Supplementary file 2 — Additional file 2: Phylogenetic analysis of the PAAR-repeat domain containing proteins. The extension sequences of the class 2 and 5 PAAR-repeat proteins in this analysis were excluded to compare the PAAR-repeat domains only, i.e. based on the class 1 PAAR-repeat proteins. The A. baylyi APD1 PAAR-repeat proteins have been included for comparative purposes. The proteins originate from the following strains; _WM99c, WM99c; A1S_, ATCC 17978; HMPREF0010_, ATCC 19606T; ACIAD, ADP1; ACICU_, ACICU; AB57_, AB0057; ABSDF, SDF; and _6870155, 6870155. The different colors indicate the class to which the highlighted clade belongs, class 1, 2 or 5 as described previously [60]. (TIFF 6 MB) [file 12864_2014_6711_MOESM2_ESM.tiff]
